# Using community-based participatory research methods to build the foundation for an equitable integrated health data system within a Canadian urban context

**DOI:** 10.1186/s12939-024-02179-3

**Published:** 2024-07-01

**Authors:** Dianne Fierheller, Casey Chu, Chelsea D’Silva, Arvind Krishendeholl, Abdul Arham, Angela Carter, Keddone Dias, Isaac Francis, Marcia Glasgow, Gurpreet Malhotra, Ian Zenlea, Laura C. Rosella

**Affiliations:** 1 The Institute for Better Health, Trillium Health Partners, Mississauga, ON Canada; 2 Roots Community Services Inc, Brampton, ON Canada; 3 UMass Chan-Baystate Medical Centre, Springfield, MA USA; 4 LAMP Community Health Centre, Etobicoke, ON Canada; 5 University of Toronto, Mississauga, ON Canada; 6 William Osler Health System, Brampton, ON Canada; 7 Indus Community Services, Mississauga, ON Canada; 8 University of Toronto, Toronto, ON Canada

**Keywords:** Health Equity, Data, Community-based Participatory Research, Data Justice, Equity-Deserving communities, Racialized populations or communities

## Abstract

Health inequalities amplified by the COVID-19 pandemic have disproportionately affected racialized and equity-deserving communities across Canada. In the Municipality of Peel, existing data, while limited, illustrates that individuals from racialized and equity-deserving communities continue to suffer, receive delayed care, and die prematurely. In response to these troubling statistics, grassroots community advocacy has called on health systems leaders in Peel to work with community and non-profit organizations to address the critical data and infrastructure gaps that hinder addressing the social determinants of health in the region. To support these advocacy efforts, we used a community-based participatory research approach to understand how we might build a data collection ecosystem across sectors, alongside community residents and service providers, to accurately capture the data about the social determinants of health. This approach involved developing a community engagement council, defining the problem with the community, mapping what data is actively collected and what is excluded, and understanding experiences of sociodemographic data collection from community members and service providers. Guided by community voices, our study focused on sociodemographic data collection in the primary care context and identified which service providers use and collect these data, how data are used in their work, the facilitators and barriers to data use and collection. Additionally, we gained insight into how sociodemographic data collection could be respectful, safe, and properly governed from the perspectives of community members. From this study, we identify a set of eight recommendations for sociodemographic data collection and highlight limitations. This foundational community-based work will inform future research in establishing data governance in partnership with diverse and equity-deserving communities.

**Supplementary Information:**

The online version contains supplementary material available at 10.1186/s12939-024-02179-3.

## Background

Systemic discrimination is ingrained within social systems and institutions worldwide, reaching all sectors, including healthcare [1, 2]. Health system design, delivery and evaluation have a long history of systemic racism and discrimination, often targeting, harming, and further marginalizing racialized and equity-deserving communities [1, 3]. In Canada, these practices can lead to delayed care and premature death among individuals from racialized and equity-deserving communities [4, 5]. The lack of appropriate sociodemographic data collection (that encompasses socioeconomic and identity-related information) contributes to widening health inequities by hindering health systems’ and community organizations’ abilities to understand and act on health disparities associated with sociodemographic factors. Intersectional data that capture an individual’s unique health and social needs, such as race, ethnicity, language, gender identity, age, employment, and housing, are required to create meaningful and culturally specific service provision and support for diverse communities [6].

Within the primary care setting, collecting sociodemographic data has been cited as a feasible and worthwhile process valued by patients [7–9]. However, many individuals, particularly those from racialized and equity-deserving communities, have reported experiencing vulnerability and discomfort when having their personal information collected [10]. Some individuals worry that the data collected could be misused or used in a discriminatory way to negatively impact their care [10]. Therefore, it is necessary to develop ethical and safer sociodemographic data collection processes that reduce harm. This action requires drawing on the principles of the data justice movement, which believes “historical (and ongoing) ways of collecting and sharing data […] erase, invisiblize, misrepresent, or harm marginalized communities” [11] and emphasizes prioritizing community interest and participation to prevent further harm and marginalization [12]. Data justice principles recommend that data are intentionally collected to serve individuals and communities and promote community self-determination. The principles advocate that collection processes be mindful of the emotional impact experienced by those providing personal and sensitive information, that data are centred around community needs, preferences and priorities and that once provided, personal data requires ethical care, which includes ensuring privacy and security [3, 13–17].

Internationally, the World Health Organization recommends conceptualizing data and data collection from social justice and human rights perspectives with principles of participation, data disaggregation, self-identification, transparency, privacy, and accountability to address racial discrimination, promote intercultural health services and reduce health inequities [2, 18]. In Canada, there have been repeated calls for the collection of race-based data as a way to address health and social disparities resulting from systemic discrimination. Such standards include the Black Health Equity Working Group’s Engagement, Governance, Access, and Protection (EGAP) framework, the First Nations Information Governance Centre’s Ownership, Control, Access, and Possession (OCAP) principles, the Canadian Institute for Health Information (CIHI)’s guidelines related to race-based data collection in healthcare settings, and British Columbia’s Office of the Human Rights Commissioner’s framework for the collection of race-based, Indigenous and other disaggregated data for addressing systemic discrimination, from their report: “Disaggregated demographic data collection in British Columbia: The grandmother perspective”. Each of these highlight the importance of governance, community engagement, safety, individual and collective ownership, respectful relationships, and proper training for collecting data [19–21]. While there have been numerous calls to action and recommendations to transform data collection in healthcare, there have been few reported examples of how these recommendations have been developed at a regional level within the health system and alongside the diverse communities from which data are collected.

Our team of health system researchers, community organization leaders and community members began this project with the understanding that community members needed to be involved in co-designing the development of safe and culturally sensitive data collection processes and tools. Drawing on data justice principles, we wanted to ensure that historically excluded voices of diverse communities in the regional municipality of Peel, Ontario, Canada, were included. Therefore, we used a community-based participatory research (CBPR) approach – a model of research that partners with communities on all phases of research – and population health analytics to understand current gaps and opportunities in sociodemographic data collection in Peel. This approach ensured meaningful community engagement and integrated academic and community-based knowledge throughout the research process [22, 23]. CBPR emphasizes community-driven and social-action-oriented principles [24] and allows the project to continue honouring and incorporating community knowledge, experiences and voices through shared learning, co-creating knowledge and capacity building [25].

In this study, the community is centred within every step of the research process, including defining the aims and methodologies used and interpreting, sharing and acting on findings. Our project aimed to identify the factors needed to operationalize community and healthcare data collection and to understand the barriers and facilitators of culturally safe, trauma-informed and effective sociodemographic data collection. This paper demonstrates the successful application of CBPR principles to explore the gaps and opportunities for building a system for collecting sociodemographic information in the health system.

## Methods

### Setting and context

This study takes place in the regional municipality of Peel (consisting of the municipalities of Mississauga, Brampton, and Caledon) in Ontario, Canada. Peel has approximately 1.38 million residents, with 52.2% and 43.0% of the population living in the suburban cities of Mississauga and Brampton, respectively, and 4.8% residing in the rural Town of Caledon [26]. The Peel region is one of the most diverse in Canada, with approximately 60% of the population identifying as a visible minority – 81% in Brampton, 61% in Mississauga, and 33% in Caledon [27]. This diversity makes Peel a microcosm for other urban Canadian communities and the ideal setting for innovative approaches that could be replicated in other communities across Canada and globally. Peel region faces serious health inequities that disproportionately affect marginalized communities. Existing research and limited data from the region reveal a greater burden of chronic conditions and premature mortality among neighbourhoods with lower socioeconomic status, as well as severe challenges for managing chronic conditions among marginalized groups [29–31]. These were amplified by the COVID-19 pandemic. At one point during the pandemic, Brampton reportedly had one of the highest rates of COVID-19 in Ontario; in one neighbourhood, nearly one in five tests were positive – Peel was considered one of the hardest-hit areas in the country [28].

In February 2021, the Anti-Black Racism & Systemic Discrimination Healthcare Collective (ABR-SDHC), a group of community and health service providers from local organizations, committed to meeting the health and social service needs of racialized communities in Peel, Halton and the Greater Toronto Area, made a call to action to health system leaders through a position paper entitled “*The Outcomes of Oppressive Systems and a Collective Call to Co-Design an Equitable and Inclusive Health System in Peel*” [4]. The report aimed to engage with health systems leaders, including Trillium Health Partners (THP), Canada’s largest community hospital system, serving over one million of Canada’s most ethnically diverse communities. In a dialogue about anti-Black racism and systemic discrimination with THP leadership and Peel community leaders, one of the most pressing issues raised was the critical data and infrastructure gaps that severely hindered Peel’s action to address the social determinants of health (SDOH). In the context of the pandemic, while there was some data on communities that tested positive for COVID-19, little was known about which communities were hardest hit by outcomes such as hospitalizations and deaths [29]. This gap in race-based data made it challenging to develop specialized public health initiatives and resources. To address the sociodemographic data gap, Peel community leaders advocated to the Ministry of Health and the regional public health unit to collect race-based data that they believed was vital to identifying and supporting impacted communities in the region [4, 30].

### Design

We describe the steps of our CBPR project, including (1) Developing a Community Engagement Council (CEC) (2) Defining the Problem with the Community (3) Mapping What Data is Actively Collected and What is Excluded (4) Understanding Experiences of Sociodemographic Data Collection (5) Analysis and Results (6) Acting on Findings.

### Study steps

**Step 1: Developing a Community Engagement Council.** We established a CEC comprising community members, organizational leaders, and researchers. The CEC represented diverse perspectives from across the Peel region, including those identified from racialized and non-racialized communities, newcomers and long-term residents, youth and seniors. The CEC established a project governance structure and principles of collaboration to address power differentials within the team based on individual identities such as profession, education, health, gender, race, ethnicity, age, poverty, and ability [22, 23, 31]. An output of this process was a Terms of Reference that outlined the role and responsibilities of the CEC and project members [see Additional file 1]. The CEC met virtually once a month for one hour to guide all project activities, including: refining the objectives and scope of the project, co-designing the survey and workshops, analysis, and interpretation; identifying critical issues for action and strategies for the next steps; planning the dissemination of project findings, and evaluating study processes and knowledge translation.

**Step 2: Defining the Problem with the Community.** As the project’s objectives were conceptualized broadly, early conversations with the CEC focused on co-identifying the key health inequity priorities in Peel that built on the existing work related to data and health equity. Through ongoing discussions, the members collectively identified issues related to Peel residents’ access to primary care providers as the project’s focus. Access to primary care services has been a point of discussion in the Peel region for decades [32] and community partners were concerned about residents’ access to primary care providers during the COVID-19 pandemic. The problem statement for the project, therefore, became: *How can sociodemographic data inform who is and who is not accessing primary care services in the Peel region?* Once primary care services were identified as the healthcare setting of focus, the study team engaged with multiple primary care networks, including family practice organizations (e.g., the Family Health Teams model in Ontario) and research teams working on primary care data collection, to understand the current state of sociodemographic data collection in primary care and gain insight on how this project can engage with primary care providers to collect data.

**Step 3: Survey to Map the Data Collected and Excluded.** To work towards building a more equitable data system for primary healthcare in Peel, we first needed to understand what the data system currently looked like, what data exist, how data are collected, what data are being collected and what data are missing. To answer these questions about the current data system, we worked with members of the CEC to co-develop and conduct a data gaps survey. This survey served to gather information about the current landscape of sociodemographic data collection in community organizations, health systems, and primary care settings. In addition, the survey was used to gather information on what specific variables were being collected, their format and their use. The United Nations Development Programme’s Guide to Data Innovation [33] was used as a framework for survey design in discussions between the study team and the CEC. The online survey took 10–15 min to complete and contained multiple-choice, checkboxes, and open-ended questions [see Additional file 2 for survey questions]. The CEC, community organizational leaders, primary care leaders and provider networks (from step 2) helped disseminate the survey to providers. Leaders from community organizations, health systems and primary care settings were asked to complete the survey on behalf of their practice or organization. The survey started by recording the participant’s role and workplace. Then, it contained general questions about how they collect sociodemographic information, in what format the data are recorded, the method of data collection, and which sociodemographic indicators are collected. The study team and CEC disseminated the survey throughout primary care and community organization leader networks.

**Step 4: Understanding experiences of sociodemographic data collection.** To continue building our understanding of the current data system, we conducted workshops with community members and service providers to understand their experiences with sociodemographic data gathering. The CEC and research team co-designed several virtual workshops to engage service providers and community members in discussing sociodemographic data collection in primary care settings. Both workshops were designed to encourage engagement through group discussion, using a Mentimeter© (an online interactive polling tool), break-out rooms with a facilitator and note taker, and reporting back to the larger group. Informed by the CEC, it was decided that we would not audio-record workshop sessions to create a safe space for participants to share their experiences. Participants were recruited by the CEC and research team by emailing workshop registration details to primary care and community organizational networks in the Peel region.

***Service Provider Workshops.*** We invited community organization leaders, primary care leaders, health system leaders and service providers working in primary care in Peel to two 90-minute workshops. The main objectives were to (1) validate the problem statement and (2) discuss the barriers and facilitators to collecting sociodemographic data in primary care. The CEC and research team members facilitated the workshops and note-takers were also assigned to each break-out room discussion to capture the discussion.

***Community Member Workshop.*** We invited Peel community members to one 90-minute workshop. The workshop’s main objective was to ask community members about their preferences and experiences of being asked about sociodemographic data. Similar to the service provider workshops, facilitators included members of the research team and CEC members representing community voices and perspectives. Note-takers were also assigned to each break-out room discussion to capture the discussion.

**Step 5: Analysis and Results.** The results from the survey and workshops were analyzed separately, with the involvement of the CEC and their input, and findings were synthesized at the end. First, the survey responses were analyzed descriptively, looking at the proportions of participants who chose an option or summarizing content from free-text form response. These results were presented to the CEC and their reactions and feedback were noted. Second, the workshop notes were analyzed using conventional content analysis by two members of the research team and one CEC member [34]. Data were reviewed word by word to derive codes that captured critical thoughts or concepts. Excerpts from the notes were grouped and organized under the codes that best represented the data. After reading the excerpts within codes, related codes were organized to create meaningful categories [35]. These preliminary categories were presented and refined by the CEC and ABR-SDHC, some of whom also attended the workshops and were able to ensure that the categories represented the workshops. This iterative and participatory approach allowed for further refinement of the category descriptions. Lastly, the research team, the CEC and the ABR-SDHC discussed the categories and survey results to co-design recommendations for action.

**Step 6: Acting on Findings.** An essential aspect of CBPR is collaboratively deciding how to act on the findings [36]. Through an integrated knowledge exchange approach, the objectives, methods, and knowledge translation outputs were co-developed with members of the ABR-SDHC, researchers, community organizations and community members through ongoing CEC meetings. This included co-writing a report for the community on findings and recommendations titled: *We Are All Accountable: Collective Action Through Data to Co-design a More Equitable and Integrated Health System in Peel Region* to share with the community and serve as an advocacy tool (https://familyandchildhealth.ca/wp-content/uploads/2023/08/Study-Final-Report-2023-1.pdf). Through ongoing discussions with the CEC and ABR-SDHC, we decided to organize a Peel Health Equity forum, which served as a platform to workshop findings and establish plans of action emerging from recommendations with health systems and community leaders.

## Results

We successfully established a CEC that included six community members, three community organizational leaders, and three research team members. The CEC represented diversity across agencies and within the community to ensure a variety of perspectives were incorporated throughout the project. Findings from our project are summarized below.

### Survey

The survey was distributed to 150 people through members of the CEC, primary care and community organizational leaders, and provider workshop participants. We received a total of 21 responses for the survey, each representing separate community organizations, health systems and primary care settings. Out of all respondents, 16 used sociodemographic data in their day-to-day work. Of the respondents, 13 were from community organizations or the health system/hospital (11 using sociodemographic data). Eight respondents were primary care providers (5 using sociodemographic data). Most respondents reported using an electronic medical record (EMR) to collect sociodemographic data, with only a few using paper to collect this information.

Most respondents reported collecting sociodemographic data during patient interviews and while taking a history. Only one of the five primary care provider respondents who reported using sociodemographic data collected gender identity, ethnicity, religion, income and preferred language. To some degree, all community organization respondents collected the sociodemographic data types listed on the survey. Specifically, all respondents collected sex, age, gender identity, ethnicity, race and preferred language (*n* = 10 for each). To a slightly lesser extent, respondents collected address (*n* = 9), email address (*n* = 9), immigrant status (*n* = 9) and marital status (*n* = 8). Additional data that respondents filled in included child custody, who lives with the child (from pediatrics provider respondents) and dietary requirements and restrictions.

For primary care providers, sociodemographic data were mainly used by clinicians and, to a lesser degree, nurses, allied health professionals, practice managers and administrative staff. For community organizations, sociodemographic data were used by case workers, intake workers and client navigators, service or organization managers, social workers and counsellors or therapists (less often by healthcare professionals or administrative staff).

### Community and provider workshops

We held three workshops, with 44 community members, six primary care providers, and 20 community service providers. Participants highlighted three key considerations for sociodemographic data collection.

**Safety and governance.** Participants emphasized the importance of having a clear and justified purpose for data collection and sharing this information with service users when collecting data. This would encourage providers to collect data and service users to provide data. Participants also suggested that the community should govern data usage and storage; data protection must be transparent.

**Tailoring approaches**. Participants noted that data collection approaches should be tailored to who is collecting data and the preferences of whom data are being collected from. Factors to consider include the timing of data collection (i.e., longitudinal data collection rather than trying to collect all the data at a single time point and asking at appropriate time points) and using appropriate language (i.e., accessible and respectful language).

**How should data be used?** We also asked about desired reasons for collection to both community members and providers. Both groups agreed that data should be used to inform clinical care. Providers underscored the importance of using these data to inform program or service delivery and to be used to report to the government or funders. On the other hand, community members were comfortable with their sociodemographic data being used for quality improvement, research, and health service planning.

### Co-designed recommendations for building sociodemographic data infrastructure

Throughout the survey and community workshops, participants directly offered suggestions on proper sociodemographic data collection. These included: having staff with training to collect the data; acknowledging and creating funding for the time it takes to collect sociodemographic data and training needed; analytic resources to collect, clean, and extract data for use; standardized electronic medical record classifications to enable data sharing and linkage; leadership buy-in; a community-driven and transparent purpose for the collected data; accurate ethnicity data that contributes to health equity for racialized and marginalized people.

High-level recommendations based on the survey and workshop results, as well as input from the CEC and ABR-SDHC, are shown in Table 1.

**Table 1:** Recommendations on collecting integrated sociodemographic data within the health system

1. Collectors need to provide a well-established purpose and rationale before collecting data. How the sociodemographic data will be protected and secured should be transparently communicated. 2. Collectors should flexibly offer multiple modes of data collection to ensure accessibility for all. 3. Collectors should engage in collection methods that ensure confidentiality is observed and respected. 4. A transparent and robust data governance structure is needed before data collection.
5. Data collection should be standardized where possible and collected in a way that ensures interoperability with other data systems. 6. Sociodemographic data should be collected in a consistent format and accessible to service providers across care settings. 7. System planning for data sharing should consider including centralized data collection. 8. Training and broader education are needed to include an anti-oppressive/anti-racist approach to support practitioners collecting data to understand how positions of power based on race, ethnicity, class, gender, age, ability etc. impact patient and service provider relationships and experiences with sociodemographic data collection.

## Discussion

We applied a CBPR approach to understanding how we could collect sociodemographic data for the health system in a large urban Canadian region by incorporating feedback from multiple stakeholders. A critical aspect of this approach involved recruiting a CEC to work alongside the research team at each stage of the research process. Ongoing community engagement and participation allowed us to focus on a community-identified priority area – data collection in primary care – which the research team may not have considered in isolation. We collaborated at regular monthly CEC meetings to co-design two data collection methods. First, we designed and administered a survey to gain insight into the current landscape of sociodemographic data collection in Peel. We then designed and hosted workshops with community members and service providers to understand critical considerations surrounding sociodemographic data collection, which could impact how we design a data ecosystem in primary care. We found that primary care providers seldom collect sociodemographic data from service users and rarely from an intersectional perspective. In contrast, community organizations collect various sociodemographic data from service users, and providers use this information to deliver tailored support and services. Community input collected throughout the project suggests that if primary care providers were to expand sociodemographic data collection, several factors need to be accounted for, including transparency around the purpose of data collection, data use, and data storage. Additionally, it is critical to appropriately match the mode of data to the setting and individuals’ level of comfort. Our results also noted several appropriate ways to use these data (e.g., clinical care and quality improvement).

The barriers we discovered in our results related to sociodemographic data collection are supported by a previous study by Yoon & Copeland (2020), which found that while data collection is helpful to community-based organizations, there are numerous challenges in accessing the data [37]. Our findings also align with Canadian sociodemographic data collection guidelines, such as the Engagement, Governance, Access, and Protection (EGAP) framework, the Ownership, Control, Access, and Possession (OCAP) principles, and the Canadian Institute for Health Information (CIHI)’s guidelines for race-based data collection [19, 20], where there was strong emphasis on themes of data governance, community engagement, safety, individual and collective ownership, and proper training. Our results also reveal the importance of transparency in data collection, which echoes British Columbia’s Office of the Human Rights Commissioner framework stressing that data collection should serve as a tool for making systemic inequities more visible and be done with a clearly defined process and purpose [22]. Additionally, we expand on existing work that outlines potential harms and ethical considerations when collecting sociodemographic data in a healthcare setting [10] by moving beyond highlighting concerns such as privacy and misuse to creating a deeper understanding of how we can support trust-building within the sociodemographic data collection process. For example, we found that community members would feel more comfortable sharing sociodemographic information if it was clear how the data would be used and if the mode of data collection was tailored to individuals’ preferences. Lastly, we gained an understanding of the current state of sociodemographic data collection in primary care in the Peel region (i.e., who collects it and how), which provides a framework in which we can apply our findings to design a suitable data collection infrastructure in Peel that integrates community data into primary care data.

There is a long history of sociodemographic data being collected and used in harmful ways within health research and systems [3]. Building on the advocacy of data justice scholars and community leaders, we argue that bringing CBPR methodology alongside data science is vital to ensuring community needs and priorities are centred on building and maintaining data collection processes and systems [38]. Engaging diverse community perspectives in health system design can increase validity, support the implementation of services through partnerships and help design effective and meaningful systems and services [22–25]. Community members of the ABR-SDHC in Peel argued in their position paper for health system leaders to re-evaluate how and why data are collected within the current health system and how data are used to inform decision-making [4]. The collective also advocated for community voices to be included in co-designing data collection processes. This project was the first step in learning about how sociodemographic data could be collected more safely and builds on the work of data justice scholars, which draws on various fields, including Indigenous data sovereignty [3] and Black feminism [38]. A CBPR approach allowed the project to include ongoing community engagement as we worked to centre and prioritize the needs and experiences of equity-deserving community members. Shah & Sekalala (2023) further argue the importance of “equitable participation” that is required when building and organizing data systems within and across health spaces [38]. Our CBPR methodological approach allowed us to practically apply the recommendations of health data justice scholars alongside diverse communities in Peel. Throughout each project stage, opportunities were created for individuals from diverse communities across Peel to be central to the project through CEC participation, workshop engagement, analysis of findings and knowledge translation.

### Limitations

We want to note three main limitations of our research. First, we recognize that the response rate for our data gaps survey may appear to be low, but the aim of our dissemination plan was to have a representative from each entity (community organization, health system, or primary care organization) complete the survey. This plan was informed by our engagement meetings with primary care leaders in our region before and during our project, who notified our team that primary care providers were overburdened, and few had the bandwidth to participate in research activities. Our survey respondents were representatives and leaders of provider organizations, which have extensive and holistic knowledge of the critical barriers to sociodemographic data collection in primary care delivery. Second, we acknowledge that while we strived to include diverse representation and perspectives, more work is needed to ensure that we continue to centre those who identify from Indigenous, Black, African and Caribbean, South Asian and racialized communities as we move into the future phases of our work. To do this, we argue that future research in building an integrated data ecosystem that includes community and healthcare data must use CBPR approaches that position diverse community voices at the centre. Lastly, we note that CBPR approaches are inherently context-specific, and our work may be limited in generalizability to other contexts. However, we argue that the steps we took can be applied to work on health data infrastructure development in many contexts, especially among urban and diverse municipalities.

## Conclusions

Historically, healthcare systems, including data collection processes, have been influenced by systemic racism, inflicting harm on racialized and equity-deserving communities [3]. Many healthcare institutions are trying to understand how they might redesign institutional practices, tools and infrastructures through anti-racist and anti-oppressive ways. Yet, these institutions continue to fall short when trying to move these social justice and health equity frameworks into practice. While there are many calls to collect sociodemographic and, specifically, race-based, data to improve equity and health system accountability, more advocacy is needed on its value and importance by health care institutions and providers. There also needs to be more meaningful engagement with diverse communities on how best to do this to minimize harm and maximize benefit in terms of reducing health inequities. This study fills an important gap in understanding the perspectives of the communities impacted and whose data would be collected and used. This CBPR project exemplifies how data justice recommendations can be carried out alongside community partners in meaningful and ethical ways. It also demonstrates that ongoing community engagement and co-design are possible. Future work will explore co-designing data governance and standardizing the data ecosystem with community residents and partners in the region of Peel.

### Electronic supplementary material

Below is the link to the electronic supplementary material.

Supplementary Material 1. Additional file 1.Pdf: Community Engagement Council – Project Terms of Reference.

Supplementary Material 2. Additional file 2.Pdf: Data Gaps Survey.

## Data Availability

No datasets were generated or analysed during the current study.

## Abbreviations

CBPR: Community–Based Participatory Research
EGAP: Engagement, Governance, Access, and Protection
CIHI: Canadian Institute for Health Information
ABR: SDHC–Anti–Black Racism & Systemic Discrimination Healthcare Collective
SDOH: Social Determinants of Health
CEC: Community Engagement Council
EMR: Electronic Medical Record
