# Supplementary material for: Using community-based participatory research methods to build the foundation for an equitable integrated health data system within a Canadian urban context
Source: Int J Equity Health. 2024 Jul 1;23:131. doi: 10.1186/s12939-024-02179-3 (PMC11218066; doi:10.1186/s12939-024-02179-3)
Supplement: Supplementary file 1 — Supplementary Material 1. Additional file 1.Pdf: Community Engagement Council – Project Terms of Reference. [file 12939_2024_2179_MOESM1_ESM.pdf]

Additional file 1 – Terms of Reference

Community Engagement Council – Project Terms of Reference

Note: specific names were omitted and replaced with descriptions in brackets []

Background and Purpose: the Anti-Black Racism & Systemic Discrimination Healthcare Collective (ABR&SDHC), a group of community and health service providers from local organizations and individuals committed to meeting the health and social service needs of racialized communities in Peel, Halton and the GTA, made a collective call to action to the health system leaders through a position paper, "The Outcomes of Oppressive Systems and a Collective Call to Co-Design an Equitable and Inclusive Health System in Peel". The report's purpose was to engage health systems leaders, in a dialogue about anti-Black racism & systemic discrimination. One of the most pressing issues identified was that there are critical data and infrastructure gaps that severely hinder action on the social determinants of health in Peel. The Community Engagement Council (CEC) was developed to guide this work.

Project Objectives:

- To co-design a data system that integrates individual-level data collected from healthcare and community organizations
- To capture the social determinants of health from across sectors to close critical data gaps needed to address persistent and growing health inequities in the Region of Peel
- To use culturally safe and trauma-informed approaches to guide all aspects of the project

CEC Principles

- Interactive and engaged group throughout the project
- Ensure all voices are heard, valued, and all input is considered
- Respect all opinions, expertise and knowledge to create a safe and brave space
- Diversity, equity and inclusion is paramount and is the foundation of the project
- Ensure that there is academic rigor throughout the process. This will ensure integrity in data collection, analysis, interpretation and uses (e.g. data/stats should be explained clearly so that point of the findings are preserved and not misunderstood).

**Roles and Responsibilities:**
**CEC Members:** [list of names]
• Engaging with the members of the CEC;<br>• Attending meetings and reviewing material in between meetings<br>• Supporting project activities including decision making, developing co-design sessions, discussing findings; and<br>• Sharing findings.
**Research Team Members**: [list of names]
• Leading CEC Meetings<br>• Preparing ethics applications and protocols;<br>• Providing scientific guidance and oversight;<br>• Providing regular updates to the ABR&SDHC and [research hospital] leadership.<br>• Drafting data collection tools; and<br>• Leading data analysis and report writing.

Governance: The CEC is the Advisory Board for the project. The CEC will be responsible for providing regular updates to the ABR&SDHC and [list of other relevant leadership].

Decision Making:

- Where possible the CEC will seek consensus through discussion while allowing opportunities for all members to provide input.
- A vote may be required when consensus cannot be achieved through discussion.
  - For those who vote, 50% +1 majority will be required from the CEC in order to move forward with a decision. If 50%+1 majority is not reached or there is a tie, the research team will collectively make the final decision and report back to the CEC.
  - If the research team indicates "urgent" in the email, the CEC members will be required to register a vote within 72 hours.
- If a member is not able to meet the requirements, the CEC may request that the agency or individual remove themselves from the CEC. If the agency does not remove themselves, they may risk being voted off the CEC by a 50%+1 majority of the remaining members.

PROJECT SCOPE
**In Scope**
• Planning and completing co-design sessions to create the foundation for identifying the critical enabling factors to operationalize the collection of community-based and healthcare data<br>• Establishing the infrastructure necessary to house data including data governance, data interpretation and analyses, and data stewardship<br>• Engaging community members, agencies, and researchers throughout all aspects of the project<br>• Maintaining regular communication with the ABR&SDHC and [other relevant leadership]
**Out of Scope**
• Operationalizing the collection of community-based and healthcare data and the infrastructure

Records: All records will be made available to members of the team. Recordkeeping and storage will be the responsibility of the research team. Records will be stored electronically on [name of server].

Ethical Considerations: The research team will assume responsibility for submitting the protocol to Trillium Health Partners' Research Ethics Board for exemption following protocol development and before the project begins and the results presented to the CEC.

Communication and Meetings: CEC meeting frequency will vary according to the priorities of the project and scope of work, and in accordance with deliverables. Currently the CEC is meeting monthly for 1 hour.

- All members of the CEC must commit to attending 2/3 of meetings.
- All members of the CEC must commit to responding to emails within 7 business days unless unable due to illness or, vacation.
- If an "urgent" decision" is required, CEC members will commit to responding within 72 hours.

Duration: The duration of term for the CEC is from November 1 2021 until June 30 2022.

Compensation: Community members will receive the equivalent of $25/hour for their contributions to the CEC. Agencies and researchers involved in the CEC are offering in-kind support and will not receive financial compensation.

Glossary of Terms (to be expanded as needed)

Data Ecosystem – the combination of datasets and data sources, as well as the necessary practices for collecting, storing, using, analyzing, and sharing datasets.[1]

Primary Care – health services that are individuals' first point of contact for addressing non-emergency health issues. These services focus on health promotion, illness and injury prevention, and the diagnosis and treatment of illness and injury. They can offer supports beyond healthcare, such as those related to income, housing, education, and environment.[2] Some examples include family doctor practices, nurse practitioner practices, family health teams, and community health centres.

Social Determinants of Health – non-medical factors that influence health outcomes. They are the conditions in which people are born, grow, work, live, and age, and the wider set of forces and systems shaping the conditions of daily life. These forces and systems include economic policies and systems, development agendas, social norms, social policies and political systems.[3]

Healthcare Access – care that is approachable, acceptable, available, accommodating, affordable, and appropriate.[4]

Health Inequity – systematic differences in the opportunities that different groups (based on sociodemographic and economic factors) have to achieve optimal health, leading to unfair and avoidable differences in health outcomes.[5]

Cultural Safety – an approach that considers how social and historical contexts, as well as structural and interpersonal power imbalances, shape health and health care experiences. "Safety" is defined by those who receive the service, not those who provide it.[6] Cultural safety differs from:

- Cultural Awareness: An attitude that includes awareness about differences between cultures.
- Cultural Sensitivity: An attitude that recognizes the differences between cultures and that these differences are important to acknowledge in health care.
- Cultural Competency: An approach that focuses on practitioners' attaining skills, knowledge, and attitudes to work in more effective and respectful ways with Indigenous patients and people of different cultures.
- Cultural Humility: An approach to health care based on humble acknowledgement of oneself as a learner when it comes to understanding a person's experience. A life-long process of learning and being self-reflexive.

Trauma-Informed – an approach that recognizes how violence and trauma, as opposed to individual characteristics (e.g., personality traits), can lead negative health outcomes and behaviours. These approaches increase safety, control and resilience for people who are seeking services in relation to

---

[1] Source : https://online.hbs.edu/blog/post/data-ecosystem
[2] Source : https://www.canada.ca/en/health-canada/services/primary-health-care/about-primary-health-care.html#a1
[3] Source : https://www.who.int/health-topics/social-determinants-of-health#tab=tab_1
[4] Source : https://equityhealthj.biomedcentral.com/articles/10.1186/1475-9276-12-18
[5] Source : https://www.ncbi.nlm.nih.gov/books/NBK425845/
[6] Source : https://www.heretohelp.bc.ca/visions/indigenous-people-vol11/what-indigenous-cultural-safety-and-why-should-i-care-about-it

experiences of violence and/or have a history of experiencing violence. The goal of this approach is to minimize harm to the people you serve—whether or not you know their experiences of violence. Embedding trauma and violence-informed approaches into all aspects of policy and practice can create universal trauma precautions, which provide positive supports for all people. It's not about 'treating' trauma, but to minimize the potential for harm and re-traumatization, and to enhance safety, control and resilience for individuals involved.[7]

[7] Source : https://www.canada.ca/en/public-health/services/publications/health-risks-safety/trauma-violence-informed-approaches-policy-practice.html#s1
