# Supplementary material for: Using community-based participatory research methods to build the foundation for an equitable integrated health data system within a Canadian urban context
Source: Int J Equity Health. 2024 Jul 1;23:131. doi: 10.1186/s12939-024-02179-3 (PMC11218066; doi:10.1186/s12939-024-02179-3)
Supplement: Supplementary file 2 — Supplementary Material 2. Additional file 2.Pdf: Data Gaps Survey. [file 12939_2024_2179_MOESM2_ESM.pdf]

Additional file 2 – Data Gaps Survey

Primary Care Data Gaps Survey

Description:

Thank you for your interest in our survey. This is part of a project out of the Institute for Better Health (IBH; https://www.instituteforbetterhealth.com/), an embedded research institute at Trillium Health Partners titled We are all accountable: Collective action through data to co-design a more equitable and integrated health system in Peel. This project is being led by Drs. Ian Zenlea and Dianne Fierheller from the Family and Child Health Initiative (https://familyandchildhealth.ca/), Dr. Laura Rosella (Stephen Family Research Chair in Community Health at IBH), and the Anti-Black Racism and Systemic Discrimination Healthcare Collective.

This project aims to determine: How can healthcare and community organizations collectively and safely collect and integrate individual-level data that capture the social determinants of health (SDOH) to understand who does and does not access primary care in the Region of Peel? To answer this question, we have prepared this survey and series of co-design workshops with primary care partners, community organizations, health systems, and researchers in Peel.

You are being invited to take part in this survey to help us identify the current landscape of data to support healthcare in primary care settings. This survey will ask about what kind of sociodemographic data you collect or use in your work, and it will take 10 minutes.

We understand that this survey may not be accessible to some; thus, if you wish to arrange a phone call or video call to fill out this information, please contact [contact email].

Introduction (all respondents answer):

- Name (optional)
- Email (optional; note: please provide it if you would like to be contacted about the progress of this project and/or opportunities to provide further input)
- Organization Name (required)
- **[LOGIC – color-coded]** Please select the option (s) that best describes your main place of work (*MC*).
    - Family Health Team
    - Family Health Organization
    - Family Health Group
    - Walk-In Clinic
    - Solo Family Practice
    - Community Health Centre
    - Community Agency
    - Health Systems/Government Entity
    - Research Institute/University

    - Other
- Please select the option that best describes your role (*check-box*).
    - Family Doctor
    - Community Organization Leader
    - Nurse
    - Researcher
    - Health Systems Leader, Manager, Analyst
    - Health Service Administrative Staff
    - Allied Health Professional
    - Other: specify ________________________
- Does your place of work collect or use sociodemographic information (note: examples of sociodemographic information includes sex, race, gender, etc.)?
    - Yes → to applicable section (colour-coded above)
    - No
- **[If No]** Please express why your place of work does not collect or use sociodemographic information (*free form*). → END OF SURVEY

SECTION A: IF SELECTED FHT/FHO/FHG/WALK-IN/SOLO PRAC:

1. Please select the option that best describes your situation (note: examples of sociodemographic information includes sex, race, gender, etc.). My practice collects sociodemographic information using: (*MC*)
- An electronic medical record (EMR)
- Another electronic system
- A paper-based system
- A mix of electronic and paper-based

2. If applicable, please provide the name of the EMR/electronic system you use (*free form*).

3. If applicable, please describe how you use a mix of both types of systems (*free form*).

4. Please indicate which types of sociodemographic information (e.g., race, sex, gender, etc.) are collected in your organization (*check-box*)?
- Sex
- Age (e.g., date of birth, age, age ranges)
- Gender Identity
- Race (groups sharing some outward physical characteristics and some commonalities of culture and history)
- Ethnicity (groups sharing nationality, heritage, culture, ancestry, and upbringing)
- Address (e.g., postal code, city, mailing address)
- Email Address
- Immigrant Status/Citizenship

- Education Level
- Marital Status
- Religion
- Income (e.g., household income, ability to ends meet/meet basic needs, benefits, ODSB, insurance)
- Sexual Orientation
- Employment (e.g., job or employment status)
- Preferred Language (e.g., first language, language spoken at home, need for translation services)
- Household Size
- Dependents (e.g., number of people your income supports)
- Social Isolation (e.g., living alone, feeling lonely)
- Disability (e.g., severe/persistent physical and/or mental condition leading to difficulty)
- Housing Access (e.g., home ownership, long-term care facility usage, social housing)
- Medicine/Medical Supplies Access
- Phone/Internet Access
- Transportation Access (e.g., access to car or public transport)

5. For the sociodemographic information you collect, are they generally collected as discrete data or free text (*MC*)?

- **Discrete Data** (a data element that can be categorized into a classification, indicators that have a specific value and are not free text)
- **Free Text** (any type of text character, used to collect textual data such as name, address, physician notes, etc.)
- Both
- Other (please enter)

6. How is sociodemographic information typically collected at your practice (please select all that apply) (*check-box*)?

- Patient self-reported questionnaire
- Recorded by physician while interviewing patients or taking history
- Other (please enter)

6. How often are these types of information collected or asked (*check-box grid*). Please leave rows blank for information that you do not collect.

	Continuously/Ongoing	One time	N/A – not collected
Sex			

Gender… (rows below for all other types listed in #4)			

a. Please use this space for other comments about how often this information is collected (*free form*).
b. If you use other types of sociodemographic information (not listed above), please indicate the type of information and how often they are collected (*free form*).

7. Who regularly **uses** this data (*check-box grid*)? Please leave rows blank for information that you do not collect.

	Nurses	Clinicians	Allied Health Professional	Practice Managers	Office Admin	N/A – not collected
Sex						
Gender… (rows below for all other types listed in #4)						

a. If you use other types of sociodemographic information (not listed above), please indicate the type of information and who uses it (*free form*).
b. How are these data used (i.e., for patient care, planning, research, quality improvement)(*free form*)?
c. If there are other people who have access to and/or use this information, please list them (*free form)*?

9. If available, please provide any attachments (screenshots, documents, etc.) of any sociodemographic questions that you ask (*attach file - google forms)*.

10. Please provide any other comments or thoughts that did not apply to the previous questions *(free form)*.

SECTION B: IF SELECTED CHC+Community Agencies

1. Please select the option that best describes your situation (note: examples of sociodemographic information includes sex, race, gender, etc.) (*MC*) My organization collects service users' sociodemographic information using:

- An electronic system
- A paper-based system
- Both electronic and paper-based

2. If applicable, please provide the name of the electronic system you use to collect sociodemographic information on service users.

3. If applicable, please describe how both electronic and paper-based systems are used.

4. Please indicate which types of sociodemographic information (e.g., race, sex, gender, etc.) are collected in your organization (*check-box*)?
   - (options same as those in Section A #4)

5. For the sociodemographic information you collect, are they generally collected as discrete data or free text (please select all that apply) (*MC*)?
   - **Discrete Data** (a data element that can be categorized into a classification, indicators that have a specific value and are not free text)
   - **Free Text** (any type of text character, used to collect textual data such as name, address, physician notes, etc.)
   - Both
   - Other (please enter)

6. How is sociodemographic information typically collected at your place of work (please select all that apply) (*check-box*)?
   - Service user self-reported questionnaire
   - Recorded by staff member during service user encounters
   - Other (please enter)

7. How often are these types of information collected or asked (*check-box grid*). Please leave rows blank for information that you do not collect .

	Continuously/Ongoing	One time	N/A – not collected
Sex			
Gender… (rows below for all other types listed in #4)			

c. Please use this space for other comments about how often this information is collected (*free form*).
d. If you use other types of sociodemographic information (not listed above), please indicate the type of information and how often they are collected (*free form*).

6. Who regularly uses this data (*check-box grid*)? Please leave rows blank for information that you do not collect.

	Healthcare Professionals (e.g., Doctors, Allied Health)	Service Managers	Office Admin	Case Workers, Intake Workers, or Client Navigators	N/A – not collected
Sex					
Gender… (rows below for all other types listed in #4)					

d. If you use other types of sociodemographic information (not listed above), please indicate the type of information and who uses (*free form*).
e. How are these data used (i.e., for providing services, planning, research, quality improvement) (*free form*)?
f. If there are other people who have access to and/or use this information, please list them (*free form)*?

7. If available, please provide any attachments (screenshots, documents, etc.) of any sociodemographic questions that you ask (*attach file - google forms)*.

8. Please provide any other comments or thoughts that did not apply to the previous questions (*free form)*.

SECTION C: IF SELECTED HS/RESEARCH/UNIVERSITY/OTHER:

1. Please describe the population that you work with in your role (*free-form)*.

2. **[LOGIC]** Please select the option that best describes how your organization obtains sociodemographic data (note: examples of sociodemographic information includes sex, race, gender, etc.) (*MC*) My organization:

A. Collects population sociodemographic information
B. Obtains sociodemographic information from national or regional datasets (e.g., statscan, ICES, CIHI, etc.)
C. Collects **and** obtains sociodemographic information from national or regional datasets (e.g., statscan, ICES, CIHI, etc.) → **[TAKES THEM TO 2A and B]**

[if 2.A.]

3. Please indicate which types of sociodemographic information (e.g., race, sex, gender, etc.) collected in your organization (*check-box*)?
- (options same as those in Section A #4)

4. For the sociodemographic information you collect, are they generally collected as discrete data or free text (please select all that apply) (*MC*)?
- **Discrete Data** (a data element that can be categorized into a classification, indicators that have a specific value and are not free text)
- **Free Text** (any type of text character, used to collect textual data such as name, address, physician notes, etc.)
- Both
- Other (please enter)

5. How is sociodemographic information typically collected at your place of work (please select all that apply) (*check-box*)?
- Service user self-reported questionnaire
- Recorded by staff member during service user encounters
- Other (please enter)

6. How often are these types of information collected or asked (*check-box grid*). Please leave rows blank for information that you do not collect .

	Continuously/Ongoing	One time	N/A – not collected
Sex			
Gender… (rows below for all other types listed in #4)			

a. Please use this space for other comments about how often this information is collected (*free form*).
b. If you use other types of sociodemographic information (not listed above), please indicate the type of information and how often they are collected (*free form*).

7. Who regularly uses this data (*check-box grid*)? Please leave rows blank for information that you do not collect.

	Government Decision-Makers	Analysts	Researchers	Health System Leaders	N/A – not collected

Sex					
Gender… (rows below for all other types listed in #4)					

g. If you use other types of sociodemographic information (not listed above), please indicate the type of information and who uses (*free form*).
h. How are these data used (i.e., for providing services, planning, research, quality improvement) (*free form*)?
i. If there are other people who have access to and/or use this information, please list them (*free form)*?

8. Please provide any other comments or thoughts that did not apply to the previous questions *(free form)*.

[If 2.B.]
3. What is the main purpose that your organization uses sociodemographic datasets for (select all that apply)?

- Research
- Planning
- Evaluation
- Other (please indicate)

4. Please check off the name or types of the dataset(s) that your organization ***regularly uses*** for the purpose(s) listed above (please select all that apply).

- Canadian Community Health Survey
- Mental Health and Access Survey
- Census
- Tax Data
- Health Administrative Data (e.g., OHIP)
- Other (please indicate)

5. Please provide any other comments or thoughts that did not apply to the previous questions *(free form)*.

ENDING (all respondents see these questions):

Additional file 2 – Data Gaps Survey

1. What would enable you or your workplace to collect and use sociodemographic data more often *(free form)*?

2. Please provide any additional comments or thoughts that did not apply to the previous questions *(free form)*.

Thank you for participating. If you provided your email at the beginning, we may contact you to follow-up with any questions. If you have any relevant files (documents, screenshots, photos, etc.) regarding sociodemographic information collection that you would like to share with us. Please send materials to [contact email].
